# Elevated Levels of the Polo Kinase Cdc5 Override the Mec1/ATR Checkpoint in Budding Yeast by Acting at Different Steps of the Signaling Pathway

**DOI:** 10.1371/journal.pgen.1000763

**Published:** 2010-01-22

**Authors:** Roberto Antonio Donnianni, Matteo Ferrari, Federico Lazzaro, Michela Clerici, Benjamin Tamilselvan Nachimuthu, Paolo Plevani, Marco Muzi-Falconi, Achille Pellicioli

**Affiliations:** 1 Dipartimento di Scienze Biomolecolari e Biotecnologie, Universita' degli Studi di Milano, Milano, Italy; 2 Dipartimento di Biotecnologie e Bioscienze, Universita' di Milano-Bicocca, Milano, Italy; Fred Hutchinson Cancer Research Center, United States of America

## Abstract

Checkpoints are surveillance mechanisms that constitute a barrier to oncogenesis by preserving genome integrity. Loss of checkpoint function is an early event in tumorigenesis. Polo kinases (Plks) are fundamental regulators of cell cycle progression in all eukaryotes and are frequently overexpressed in tumors. Through their polo box domain, Plks target multiple substrates previously phosphorylated by CDKs and MAPKs. In response to DNA damage, Plks are temporally inhibited in order to maintain the checkpoint-dependent cell cycle block while their activity is required to silence the checkpoint response and resume cell cycle progression. Here, we report that, in budding yeast, overproduction of the Cdc5 polo kinase overrides the checkpoint signaling induced by double strand DNA breaks (DSBs), preventing the phosphorylation of several Mec1/ATR targets, including Ddc2/ATRIP, the checkpoint mediator Rad9, and the transducer kinase Rad53/CHK2. We also show that high levels of Cdc5 slow down DSB processing in a Rad9-dependent manner, but do not prevent the binding of checkpoint factors to a single DSB. Finally, we provide evidence that Sae2, the functional ortholog of human CtIP, which regulates DSB processing and inhibits checkpoint signaling, is regulated by Cdc5. We propose that Cdc5 interferes with the checkpoint response to DSBs acting at multiple levels in the signal transduction pathway and at an early step required to resect DSB ends.

## Author Summary

Double strand DNA breaks (DSBs) are dangerous chromosomal lesions that can lead to genome rearrangements, genetic instability, and cancer if not accurately repaired. Eukaryotes activate a surveillance mechanism, called DNA damage checkpoint, to arrest cell cycle progression and facilitate DNA repair. Several factors are physically recruited to DSBs, and specific kinases phosphorylate multiple targets leading to checkpoint activation. Budding yeast is a good model system to study checkpoint, and most of the factors involved in the DSBs response were originally characterized in this organism. Using the yeast *Saccharomyces cerevisiae*, we explored the functional role of polo kinase Cdc5 in regulating the DSB–induced checkpoint. Polo kinases have been previously involved in checkpoint inactivation in all the eukaryotes, and they are frequently overexpressed in cancer cells. We found that elevated levels of Cdc5 affect the cellular response to a DSB at different steps, altering DNA processing and overriding the signal triggered by checkpoint kinases. Our findings suggest that Cdc5 likely regulates multiple factors in response to a DSB and provide a rationale for a proteome-wide screening to identify targets of polo kinases in yeast and human cells. Such information may have a practical application to design specific molecular tools for cancer therapy. Two related papers published in *PLoS Biology*—by Vidanes et al., doi:10.1371/journal.pbio.1000286, and van Vugt et al., doi:10.1371/journal.pbio.1000287—similarly investigate the phenomenon of checkpoint adaptation/overriding.

## Introduction

*Saccharomyces cerevisiae* cells suffering a double stranded DNA break (DSB) activate a robust Mec1-dependent checkpoint response when DSB ends are processed to expose single-stranded DNA (ssDNA), and progression through the cell cycle is arrested prior to anaphase. Several well conserved factors are recruited at the DSB lesion, and contribute to the activation of a signaling pathway based on sequential phosphorylation events driven by the upstream kinases Tel1/ATM and Mec1/ATR which, in turn, activate the transducer kinases Rad53/Chk2 and Chk1 [1],[2]. The checkpoint response is influenced at several levels by kinases such as CDK1, CKII and Polo-like Cdc5, all involved in promoting key events throughout an unperturbed cell cycle, supporting the notion that the cellular response to DNA damage is tightly linked to cell cycle events [3]. The intensity of the DSB-induced checkpoint response correlates to the amount of the ssDNA that is accumulated at DSB lesions [4]. 5′-to-3′ nucleolytic processing of DNA ends is dependent upon several factors, including CDK1 and the nucleases Mre11, Sae2, Dna2 and Exo1 [5]. Moreover, the checkpoint is a reversible signaling pathway which is turned off when DNA lesions are repaired, thus permitting the resumption of cell cycle progression [6]. Different types of phosphatases (Pph3, Ptc2 and Ptc3) dephosphorylate and inactivate Rad53 and other checkpoint kinase targets [7]. Further, mutations in several DNA repair genes, including *SAE2*, *KU70*/*80*, *RAD51*, *RDH54*, *SRS2*, affect the inactivation of the DSB-induced checkpoint response [7],[8]. These observations suggest that the attenuation, as well the activation, of the checkpoint pathway are related to the metabolism of DSB ends, in a way that is not yet completely understood. It is also known that the checkpoint response can be attenuated when an irreparable DNA lesion is formed in the cell, leading to adaptation to DNA damage. Checkpoint inactivation during recovery and adaptation to DNA damage is a phenomenon described also in higher eukaryotes [6]. The functional role of adaptation is not completely understood; however, it was suggested that it may be partly responsible for chromosomal rearrangements, genome instability and tumorigenesis [6],[9]. Interestingly, the well conserved family of Polo-like kinases (Plks) has been involved in checkpoint adaptation and/or recovery both in budding yeast and vertebrates [10]. Cdc5 is the only polo kinase expressed in yeast, whereas higher eukaryotes usually express three or four Plks [11]. However, only Plk1, which is the most extensively studied, is a true mitotic kinase homolog to the *Drosophila* Polo kinase [11]. In yeast, *CDC5* is an essential gene and the point mutation *cdc5*-ad (a Leucine-to-Tryptophan substitution at residue 251, within the kinase domain) causes the inability to adapt to one irreparable DSB lesion and to turn off Rad53 kinase [12],[13]. However, *cdc5*-ad cells can recover from checkpoint when the DSB is repaired, suggesting that adaptation and recovery are two genetically separate processes [14]. A corresponding *cdc5*-ad mutation in Plks has not yet been isolated in mammals; however, it was found that Plk1 depletion severely blocks checkpoint recovery and adaptation [10],[15],[16], and rapidly causes cell death in cancer cells [17],[18]. Based on the fact that the DNA damage checkpoint pathway is well conserved in all the eukaryotes, it is reasonable to expect that the functional role of Cdc5 in budding yeast and of Plk1 during adaptation (and perhaps in recovery) may be conserved. Polo-like kinases contain in the C-terminal region of the protein a polo box which mediates the interaction of Plks with substrates previously phosphorylated by CDK or MAPK kinases [19]. Indeed, Cdc5 targets multiple substrates during an unperturbed cell cycle [20] and could functionally interact with several checkpoint proteins as well. In vertebrates, polo kinases regulate the DNA damage checkpoint acting on multiple factors. They phosphorylate Claspin [21]–[24], a Chk1 kinase regulator, and the Fanconi-Anemia protein FANCM [25], promoting their degradation and checkpoint inactivation. Further, Plk1, Plk3 and Plk4 interact with and phosphorylate Chk2, the ortholog of Rad53 in human cells, likely influencing its activity [26]–[28]. Interestingly, yeast Cdc5 is phosphorylated and inhibited in a Mec1- and Rad53-dependent manner [29], and several studies indicate that in mammals Plk1 activity is inhibited by ATM/ATR-signaling in response to DNA damage [30]–[33]. Further, the DNA damage checkpoint regulates Plk1 protein stability in response to DNA damage in mitosis [34]. It was also shown that Aurora kinase A phosphorylates and re-activates Plk1 to promote recovery from DNA damage [35]. Altogether, these informations suggest that the DNA damage checkpoint inhibits Plk1, thus contributing to block cell cycle progression in response to DNA damage; however, the re-activation of Plk1 is a crucial event of a feedback regulatory loop in the inactivation of the DNA damage checkpoint during recovery and adaptation.

Therefore, the activity of Plks must be finely regulated during the DNA damage checkpoint response, and it is worth mentioning that the expression of a constitutively active Plk1 protein variant overrides the G2/M arrest induced by DNA damage [30]. Indeed, Plks are frequently overexpressed in tumor cells with uncontrolled proliferation and genome instability [36]–[39], and high level of Plk1 is predictive of a bad prognosis in several cancers [40]–[44].

To further characterize the functional link between Plks and the DNA damage checkpoint and, possibly, to understand why Plks are frequently overexpressed in cancer cells, we used budding yeast as a model system to study DNA damage related events in the presence of high levels of Cdc5.

Here, we show that overproduction of Cdc5 impairs the Mec1-signaling pathway in response to an inducible DSB lesion, altering phosphorylation of Ddc2, Rad9, Rad53 and other Mec1 targets. We also found that elevated levels of Cdc5 slow down DSB ends processing, although it does not prevent the formation of ssDNA, which triggers the recruitment of checkpoint factors. Consistently, we observed that overexpression of Cdc5 does not alter the loading of the apical Mec1 kinase checkpoint complex and recruitment of the checkpoint mediator Rad9, but surprisingly it physically interact with the checkpoint inhibitor Sae2, inducing its hyper-phosphorylation and an increased and persistent binding onto a DSB lesion.

We propose that high levels of polo kinase Cdc5 override Mec1-induced checkpoint response to DSB lesions, likely by regulating multiple factors, previously phosphorylated by CDK1, involved in both DSB processing and checkpoint signaling. Our work may represent a simple model to further understand why polo kinases are frequently overexpressed in cancer cells.

## Results/Discussion

### Elevated levels of Cdc5 override Mec1 signaling

DNA damage checkpoints represent a barrier to oncogenesis; in fact, loss of these surveillance mechanism is a characteristic of early tumor development [45]. Several evidences indicate that Plks are targets of the DNA damage checkpoint in all the eukaryotes [29]–, suggesting a functional model in which the DNA damage checkpoint inhibits Plks to maintain a cell cycle block at the metaphase to anaphase transition. Indeed, numerous cancer cells have been reported to display overexpression of Plks, and this may contribute to their transformed phenotype [36]–[39].

**Figure 1:**
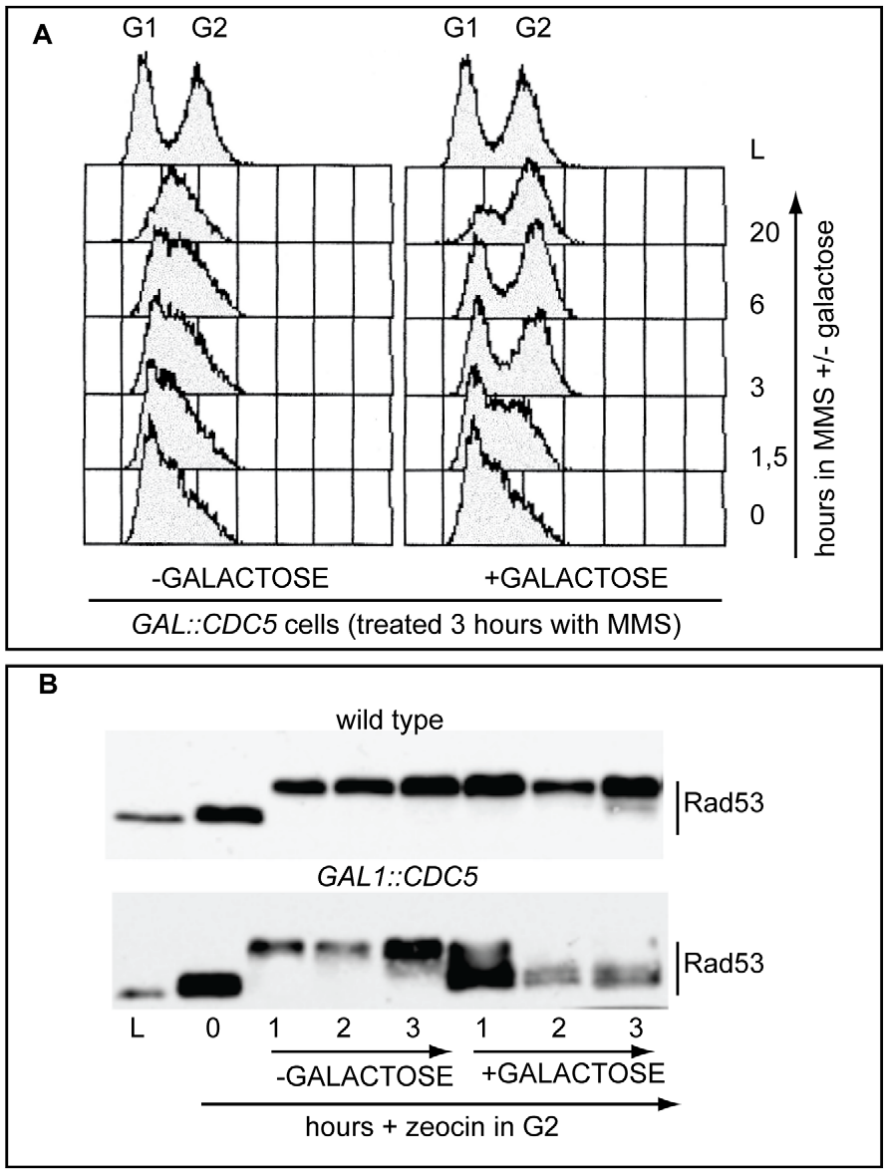
Overproduction of Cdc5 overrides the DNA replication and DNA damage checkpoints. (A) Exponentially (L) growing culture of the strain Y114 (*GAL1::CDC5*) was grown in YEP+3%raffinose and treated for 3 hours with 0.02% MMS (time 0). Then the culture is split in two and 2% galactose was added to one half, while the other half was maintained in raffinose. Samples were taken at the indicated time and analysed by FACS. (B) Cultures of the strains Y79 (wild type), Y114 (*GAL1::CDC5*), exponentially (L) growing in YEP+3%raffinose were blocked in G2/M by nocodazole treatment (0). Zeocin (50 µg/ml) was then added to cause DSBs formation and after 30 minutes of treatment, 2% galactose was added. Samples were taken at the indicated time and Rad53 protein was analyzed by western blotting with Mab.EL7 antibody.

In budding yeast, overproduction of the polo kinase Cdc5 in *cdc13-1* mutant cells with uncapped telomeres has been reported to override the checkpoint-dependent cell cycle block in the G2 phase of the cell cycle [46],[47]. We found that overproduction of Cdc5 impairs the replication checkpoint, which delays S phase in the presence of the alkylating agent MMS (methylmetane sulfonate, Figure 1A). Indeed, Figure 1A shows that MMS treated wild type cells accumulate in S phase for a very long period (1C<DNA<2C), while Cdc5 overproducing cells rapidly go through the replication phase and reach a G2/M DNA content (2C). Moreover, the DNA damage-induced phosphorylation of Rad53 is essentially abolished in Cdc5 overproducing cells treated with zeocin, an agent causing DSBs (Figure 1B).

We have to assume that, although the DNA damage checkpoint inhibits Cdc5 [29],[46], contribuiting to block cell cycle in the presence of DNA damage, when *CDC5* is placed under the control of the *GAL1* promoter, the DNA damage-induced inhibition on overproduced Cdc5 is not complete. This is likely due to the elevated Cdc5 levels, which are higher than the endogenous amount (see also Figure S1), leading to the override of the checkpoint response. Indeed, it was previously shown that the overproduction of Cdc5, which is a finely regulated protein [29], causes severe phenotypes during an unperturbed cell cycle [48]–[51].

**Figure 2:**
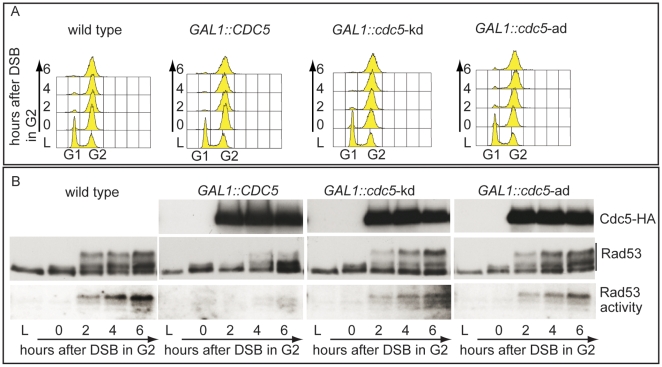
Overproduction of Cdc5 affects DSB–induced Rad53 phosphorylation and activity. (A,B) YEP+raffinose nocodazole-arrested cell cultures of wild type JKM and isogenic *GAL1::CDC5*, *GAL1::cdc5*-kd (kinase dead, K110A mutation) and *GAL1::cdc5*-ad (adaptation defective, L251W mutation) strains were transferred to nocodazole-containing YEP + raffinose + galactose (time zero). (A) Samples were taken at the indicated time points and analyzed by FACS. (B) Overproduced Cdc5 proteins have an additional HA epitope and their accumulation in galactose was analyzed by western blots using 12CA5 antibody. Rad53 was analyzed by western blots with Mab.EL7 antibodies. Rad53 in situ auto-phosphorylation activity was analyzed by ISA assay.

In order to expand the analysis on the crosstalk between polo kinases and checkpoint pathways, and possibly to understand why overexpression of Plks is often found in tumor cells characterized by uncontrolled proliferation and genome instability, we analysed the effects of elevated Cdc5 levels on the DSB-induced checkpoint cascade in *S. cerevisiae*. We took advantage of a standard yeast genetic system (JKM background) in which one irreparable DSB can be induced at the *MAT* locus by expressing the site-specific HO nuclease [8]. We overexpressed wild-type *CDC5* and the two *cdc5*-ad and *cdc5*-kd mutant alleles (adaptation-defective and kinase-dead alleles, respectively [51]) from the galactose-inducible promoter and examined Rad53 phosphorylation and *in situ* auto-phosphorylation activity, which are routinely used as markers of DNA damage checkpoint activation [52]. To prevent variations due to cell cycle differences, we first arrested cells with nocodazole in mitosis, a cell cycle stage in which the DSB-depended checkpoint can be fully activated [12], and subsequently added galactose to induce Cdc5 overproduction and HO-break formation, while maintaining the cell cycle block. Figure 2A shows the FACS profiles of the cell cultures. We observed that overproduction of Cdc5 impairs the accumulation of hyper-phosphorylated Rad53 forms and prevents Rad53 auto-phosphorylation activity in response to DSB formation (Figure 2B). Interestingly, overproduction of the protein variants Cdc5-kd or Cdc5-ad did not significantly interfere with Rad53 phosphorylation and activation, suggesting that the kinase activity of Cdc5 and its capacity to interact with specific target(s) are required to override the DSB-induced Rad53 activation.

In vertebrates, polo kinases regulate the DNA damage checkpoint response by affecting the signal transduction pathway at different levels; interestingly, Chk2, the homologue of Rad53 in human cells, interacts with and is phosphorylated by the polo kinases Plk1, Plk3 and Plk4 [26]–[28].

Therefore, we tested whether the overproduction of Cdc5 might override Rad53 activation by targeting directly the Rad53 protein and/or by acting on other upstream checkpoint factors.

We failed to co-immunoprecipitate Rad53 and Cdc5, when expressed at endogenous levels or by using the polo box of Cdc5 in a standard GST pull down assay; however, we retrieved Rad53 with overproduced Cdc5 (Figure S2). Considering such physical interaction, we analyzed how overproduction of Cdc5 might affect the events leading to full activation of Rad53, which involves a two steps-based mechanism: an *in trans* phosphorylation event mediated by PIKKs, followed by auto-phosphorylation [53]. In theory, Cdc5 might affect any of these events required to activate Rad53. We analysed the effect of Cdc5 overexpression on the PIKKs-dependent phosphorylation of Rad53 by taking advantage of the catalytically inactive *rad53*-K227A mutant. Such protein can be phosphorylated *in trans* by the upstream kinases, but does not undergo auto-phosphorylation in the presence of DNA damage [52], allowing us to separate and discriminate the two steps.

**Figure 3:**
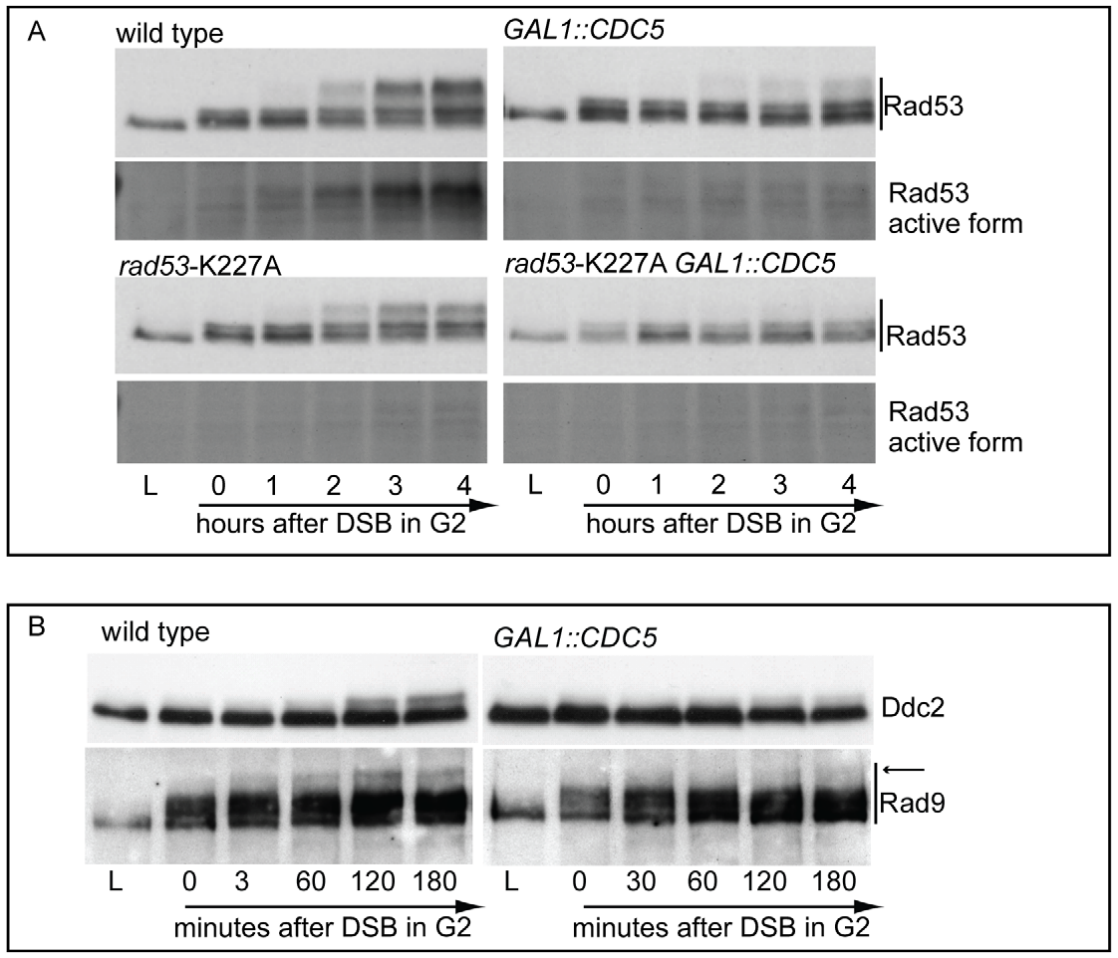
Overproduction of Cdc5 overrides Mec1 checkpoint signaling. (A) YEP+raffinose nocodazole-arrested cell cultures of wild-type JKM and isogenic *rad53*-kd (kinase dead, K227A mutation) derivative strains, with or without *GAL1::CDC5*, were transferred to nocodazole-containing YEP + raffinose + galactose (time zero). Samples were taken at the indicated time points and Rad53 was analyzed by western blots using monoclonal antibodies Mab.EL7 or Mab.F9, which recognized, respectively, all the forms of Rad53 or only the auto-phosphorylated and active forms. (B) YEP+raffinose nocodazole-arrested cell cultures of wild type JKM and isogenic *GAL1::CDC5* derivative strains, expressing *DDC2*-HA, were transferred to nocodazole-containing YEP + raffinose + galactose (time zero). Ddc2 protein was analyzed by western blots using 12CA5 antibody; Rad9 protein was analyzed by polyclonal antibodies. An arrow denotes the hyper-phosphorylation band of Rad9 accumulated specifically in response to DNA damage.

In nocodazole blocked cells, induction of a single irreparable HO cut induced Mec1-dependent phosphorylation of the Rad53-K227A protein variant (Figure 3A). As expected, the corresponding phosphorylated bands of Rad53-K227A protein were not visualized by western blot using the monoclonal antibody (Mab.F9) which is specific for the auto-phosphorylated and active Rad53 isoform [54]. Moreover, the same phospho-specific antibody did not significantly detect Rad53 in wild type cells responding to DSB when Cdc5 is overproduced, confirming the results of the *in situ* kinase assay (Figure 2B). A residual shifted band of Rad53, visualized in *CDC5* overexpressing cells through the highly sensitive Mab.EL7 antibody (both in Figure 2B and Figure 3A, and in other figures below), could reflect low levels of Rad53 activation not detected by the antibody against the active form; this is consistent with the residual Rad53 activity in the *in situ* analysis in Figure 2B. In any case, it is unlikely that this remaining Rad53 activity is sufficient to maintain a full checkpoint response, since overproduction of Cdc5 functionally overrides the cell cycle block in the presence of DNA damage.

Significantly, Cdc5 overproduction abolished DSB-induced *in trans* phosphorylation of the Rad53-K227A variant (Figure 3A). This result rules out the hypothesis that Cdc5 may override the DSB-induced checkpoint acting only on the auto-phosphorylation step of Rad53 activation, and suggests that *CDC5* overexpression likely impairs the Mec1-dependent *in trans* phosphorylation and activation of Rad53.

The residual Rad53 phosphorylation and activity in the presence of high levels of Cdc5 might suggest that the upstream Mec1 kinase, which is mainly responsible of the Rad53 activation in the presence of a single DSB in wild type cells [55], is strongly but not fully inhibited. Alternatively, Mec1 may still be functional as a kinase, but impaired in fully trans-activating Rad53. To test more directly the activity of the upstream kinase Mec1, we analysed the phosphorylation state of its interacting subunit Ddc2, the ortholog of human ATRIP, and that of the checkpoint mediator Rad9, which are known to be directly phosphorylated by Mec1 [1]. Cells were arrested with nocodazole and *CDC5* overexpression and induction of a single unrepairable DSB were induced by galactose addition (Figure 3B). Western blot analysis indicate that phosphorylated isoforms of Ddc2 and hyper-phosphorylated Rad9 (indicated by the arrow in Figure 3) accumulated after the formation of the HO cut in wild type cells, as expected; however, overexpression of Cdc5 reduced the DSB-induced hyper-phosphorylated form of both Ddc2 and Rad9, suggesting that the activity of Mec1 kinase is strongly impaired in the presence of high level of Cdc5. A careful analysis of the blot shown in Figure 3B or in analogous experiments indicates that reduced levels of phosphorylated Rad9 isoforms are present in *CDC5* overexpressing cells, suggesting that Mec1 could still retain a flebile activity toward Rad9 and Rad53, as discussed above. In addition, it is known that Rad9 is a target of multiple kinases [56] and we cannot rule out the possibility that the residual phosphorylation of Rad9 observed in cells with elevated levels of Cdc5 may be due to other kinase(-s), including Cdc5 itself.

Taken together the results shown in Figure 1, Figure 2, and Figure 3 indicate that Cdc5 activity overrides the DSB-induced checkpoint by influencing an early step of the Mec1 signaling pathway, likely reducing the functionality of Mec1 activity. However, it is possible that Cdc5 may target multiple substrates, including the Mec1 interactor Ddc2, the checkpoint mediator Rad9, whose role in promoting Mec1-to-Rad53 signaling is well established, and Rad53 itself, thus counteracting the checkpoint signaling pathway at several levels.

### High levels of Cdc5 affect DSB resection

Robust Mec1 and Rad53 activation is not triggered by the DSB itself, but requires multiple interconnected events following the formation of the lesion, including the generation of nucleolytic-dependent 5′-to-3′processing of the DNA ends and recruitment of various DNA repair and checkpoint factors onto the long stretches of the generated ssDNA [4].

**Figure 4:**
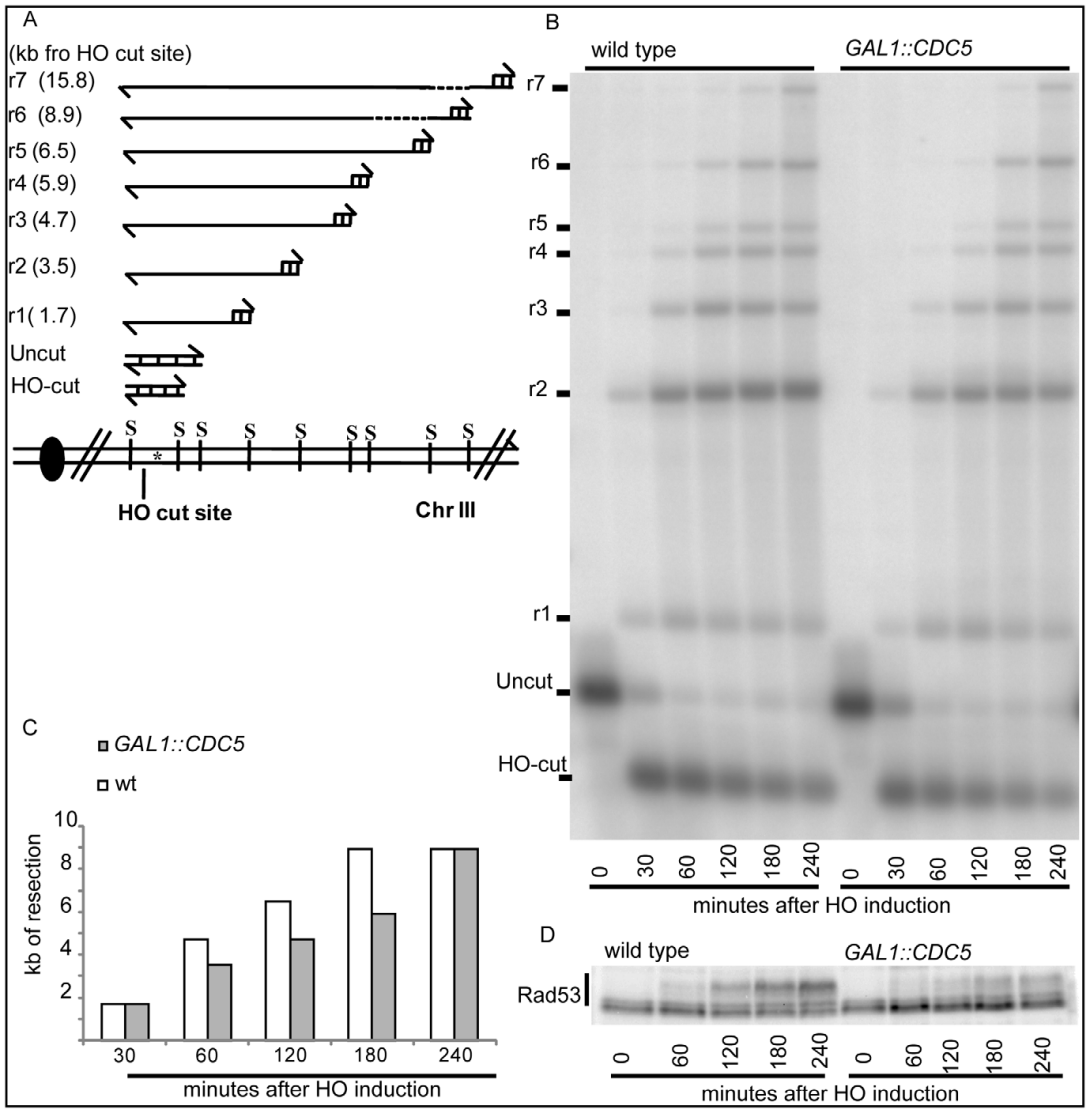
Overproduction of Cdc5 affects DSB processing. (A–D) YEP+raffinose nocodazole-arrested cell cultures of wild-type JKM MATα and isogenic *GAL1::CDC5* strain were transferred to nocodazole-containing YEP + raffinose + galactose (time zero). (A) Schematic representation of the system used to detect DSB resection. Gel blots of *SspI*-digested genomic DNA separated on alkaline agarose gel were hybridized with a single-strand RNA probe specific for the un-resected strand at the MAT locus, which shows HO-cut and uncut fragments of 0.9 and 1.1 kb, respectively. 5′-to-3′ resection progressively eliminates *SspI* sites located 1.7, 3.5, 4.7, 5.9, 6.5, 8.9, and 15.8 kb centromere-distal from the HO-cut site, producing larger *SspI* fragments (r1–r7) detected by the probe. (B) Analysis of ssDNA formation as described in (A). (C) The time of the first appearance over the background of each undigested band in the blot shown in (B) was graphically represented for both the wild type and *GAL1::CDC5* strains. (D) Western blot analysis of protein extracts with anti-Rad53 Mab.EL7 antibody.

Therefore, we investigated whether Cdc5 may control Mec1 signaling by affecting DSB processing. We measured the kinetic of ssDNA formation after a single unrepairable DSB in cells overexpressing *CDC5*. Cells were arrested in mitosis, to prevent cell cycle-dependent effects on resection [57], and samples were collected at various time points after HO nuclease induction (Figure 4). The kinetic of production of ssDNA regions in genomic DNA was tested by the loss of restriction sites distal to the HO-cut site which leads to the accumulation of undigested ssDNA fragments detectable with a strand-specific probe after alkaline electrophoresis (see the scheme of the unprocessed and processed DNA locus in Figure 4A). *CDC5* overexpressing cells reproducibly exhibited a slower DSB resection, measured by the kinetic of appearance of DNA fragments, which correlated with a reduced phosphorylation of Rad53 (Figure 4B–4D). However, we found that, although the kinetic of DSB ends resection was delayed, high levels of Cdc5 do not prevent the generation of a long ssDNA track (25 kb) which is required to repair the DSB in a specific yeast genetic background [14] by the single-strand annealing process (Figure S3).

**Figure 5:**
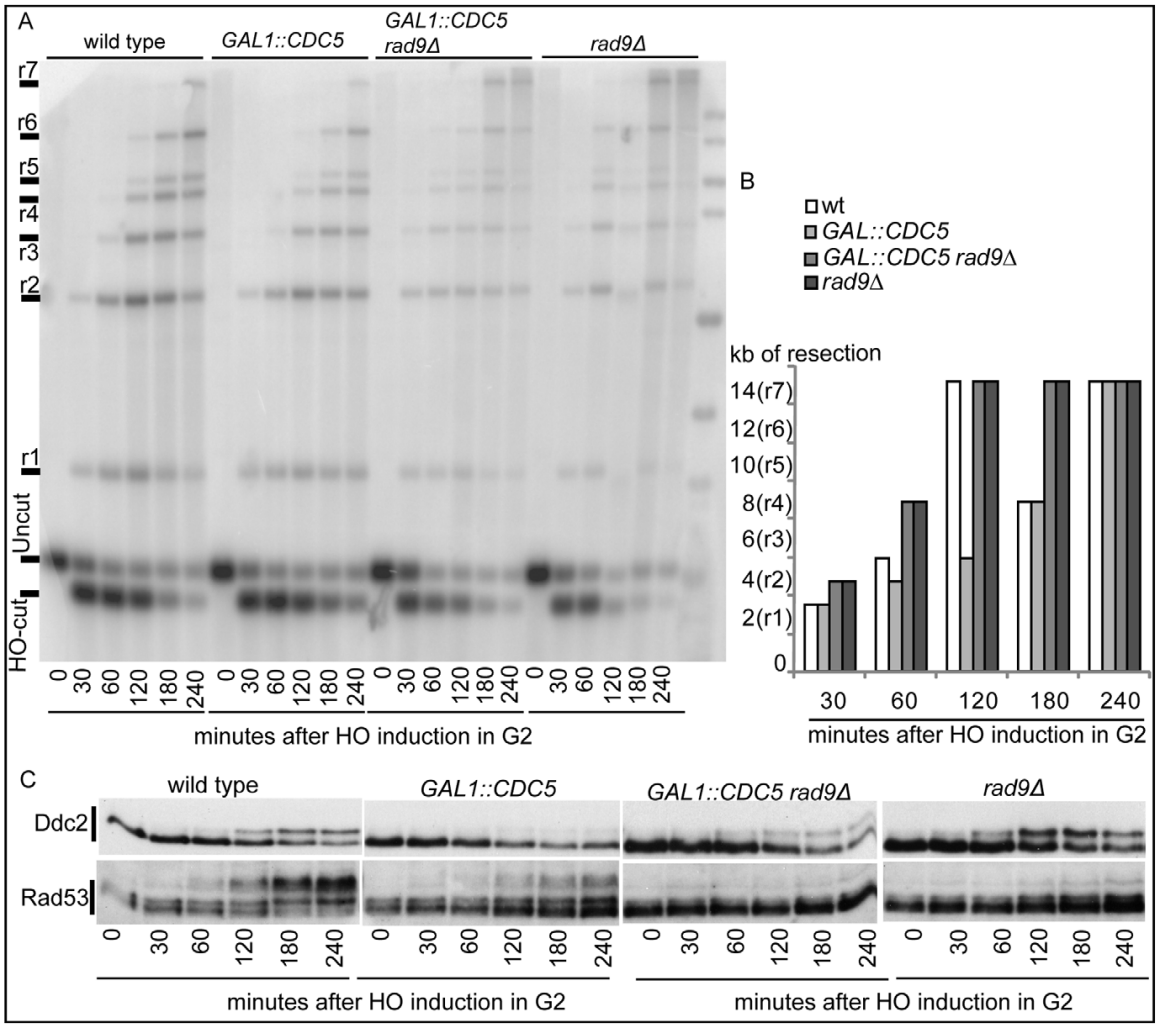
Deletion of *RAD9* gene accelerates DSB resection despites high Cdc5's levels. YEP+raffinose nocodazole-arrested cell cultures of wild type JKM MATa and isogenic *rad9*Δ strains, with or without *GAL1::CDC5*, were transferred to nocodazole-containing YEP + raffinose + galactose (time zero). (A,B) Analysis of ssDNA formation as described in Figure 4. (C) Ddc2 protein was analyzed by western blots using 12CA5 antibody; Rad53 protein was analysed by monoclonal antibody Mab.EL7.

We previously identified a role for the checkpoint mediator Rad9 in inhibiting the kinetic of DSB ends resection, likely by generating a non-permissive chromatin configuration around the DSB and/or interfering with the action of nucleases [58]. Therefore, we analyzed the Rad9 contribution in delaying DSB processing in *CDC5* overexpressing cells. Wild-type or *rad9*Δ cells, with or without *GAL1::CDC5*, were arrested in mitosis by nocodazole treatment and the same experiment described in Figure 4B was performed. We found that the kinetic of appearance of ssDNA fragments was accelerated in *rad9*Δ strains, despite the high levels of Cdc5 kinase (Figure 5A and 5B). Moreover, the faster DSB resection in *CDC5* overexpressing *rad9*Δ cells also correlated with a modest increase in Ddc2 phosphorylation (Figure 5C); however, the phosphorylated state of Ddc2 did not reach the same level found in wild-type and *rad9*Δ cells, suggesting that overproduction of Cdc5 impaired Mec1-dependent signaling also in a *rad9*Δ background. These results suggest that elevated levels of Cdc5 may slow down DSB processing through the action of the Rad9-dependent barrier on resection [58], likely targeting Rad9 itself or other factors involved in this mechanism. Interestingly, many of the proteins involved in DSB ends processing (i.e. Rad9, Dna2, Xrs2 and Sae2) are phosphorylated by CDK1 [59],[60] and inspection of their protein sequence reveals that they may be potential targets of Cdc5.

Hence, Cdc5 may influence the DSB response acting on multiple factors, affecting DSB processing and Mec1-signaling; moreover, the possibility that Cdc5 might specifically regulate Rad53 by influencing its interaction with the checkpoint mediator Rad9 cannot be excluded.

### Recruitment of checkpoint factors in *CDC5*-overexpressing cells

**Figure 6:**
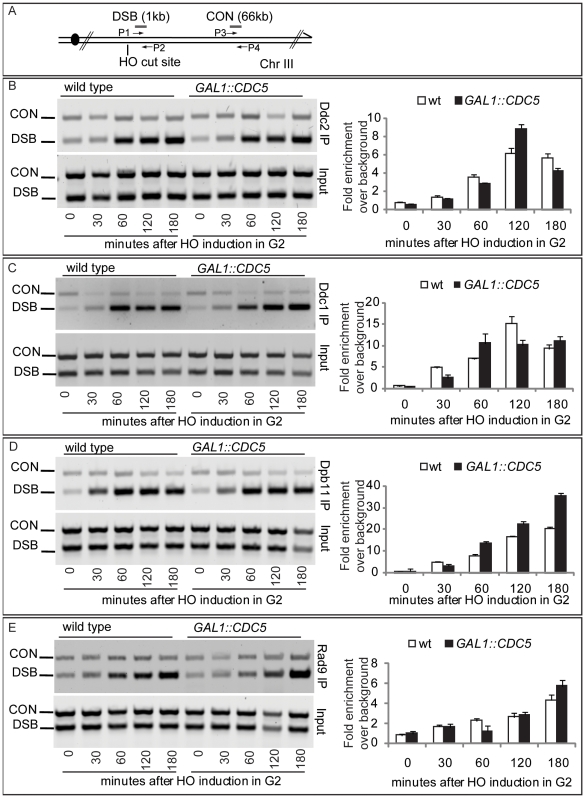
Recruitment to DSB of checkpoint factors in *CDC5*-overexpressing cells. (A) Schematic representation of the HO cleavage site with the positions of the primers used to amplify regions 1 kb (DSB) and 66 kb (CON) from the HO cut site. PCR analysis at the CON site is used as a control of background signal. (B–E) YEP+raffinose nocodazole-arrested cell cultures of wild-type JKM and isogenic *GAL1::CDC5-MYC or GAL1-CDC5-HA* strains, expressing *DDC2*-HA, *DDC1*-MYC, *DPB11*-MYC, and *RAD9*-MYC alleles, were transferred to nocodazole-containing YEP + raffinose + galactose (time zero). Cells were collected at the indicated times and then subjected to chromatin immunoprecipitation. Representative ChIP time-course analysis of protein-DSB association is shown for each protein tested before (Inputs) and after protein immunoprecipitation (IP).

Since high levels of Cdc5 did not prevent the generation of long ssDNA regions but inhibit Mec1-signaling, we tested, by chromatin immunoprecipitation (ChIP), whether overexpression of *CDC5* affected the recruitment of checkpoint factors onto the HO-induced DSB lesion in nocodazole-arrested cells. Sheared chromatin from formaldehyde crosslinked cells taken at different time-points after galactose addition was immunoprecipitated to recover checkpoint proteins (i.e. Ddc2, Ddc1, Dpb11, Rad9) carrying the MYC or HA epitope tags at their carboxyl-terminal end. Quantitative multiplex PCR was then used to monitor co-immunoprecipitation of DNA fragments located either 66 kb centromere-proximal to the *MAT* locus (CON) or 1 kb away from the HO-cut site (DSB) (Figure 6A).

Ddc2 and Ddc1 association at the DSB was not significantly affected in *CDC5* overexpressing cells blocked by nocodazole treatment (Figure 6B and 6C). The Mec1 interacting factor Ddc2 and Ddc1, one of three subunits of the stable PCNA-like 9-1-1 checkpoint complex, are recruited early onto a DSB lesion [61]–[63]. We, therefore, assume that Cdc5 overproduction does not prevent the recruitment of upstream checkpoint protein complexes onto damaged DNA. This observation also confirms that elevated levels of Cdc5, while delaying resection, do not prevent the generation of ssDNA (see Figure 4, Figure 5, and Figure S3) which is required for the recruitment of checkpoint factors [4].

Similarly, we found that overproduction of Cdc5 did not prevent the localization near the DSB of Dpb11 (Figure 6D), the yeast ortholog of TopBP1, which, together with the 9-1-1 complex, stimulates the Mec1 kinase activity [64].

Moreover, when we tested by ChIP analysis the binding of the checkpoint mediator Rad9, we found that also its localization onto the DSB was not altered in *CDC5* overexpressing cells (Figure 6E).

Taken together, the ChIP analyses of checkpoint factors at a DSB site indicate that high levels of Cdc5 kinase do not significantly interfere with the binding of checkpoint proteins to a processed DSB.

**Figure 7:**
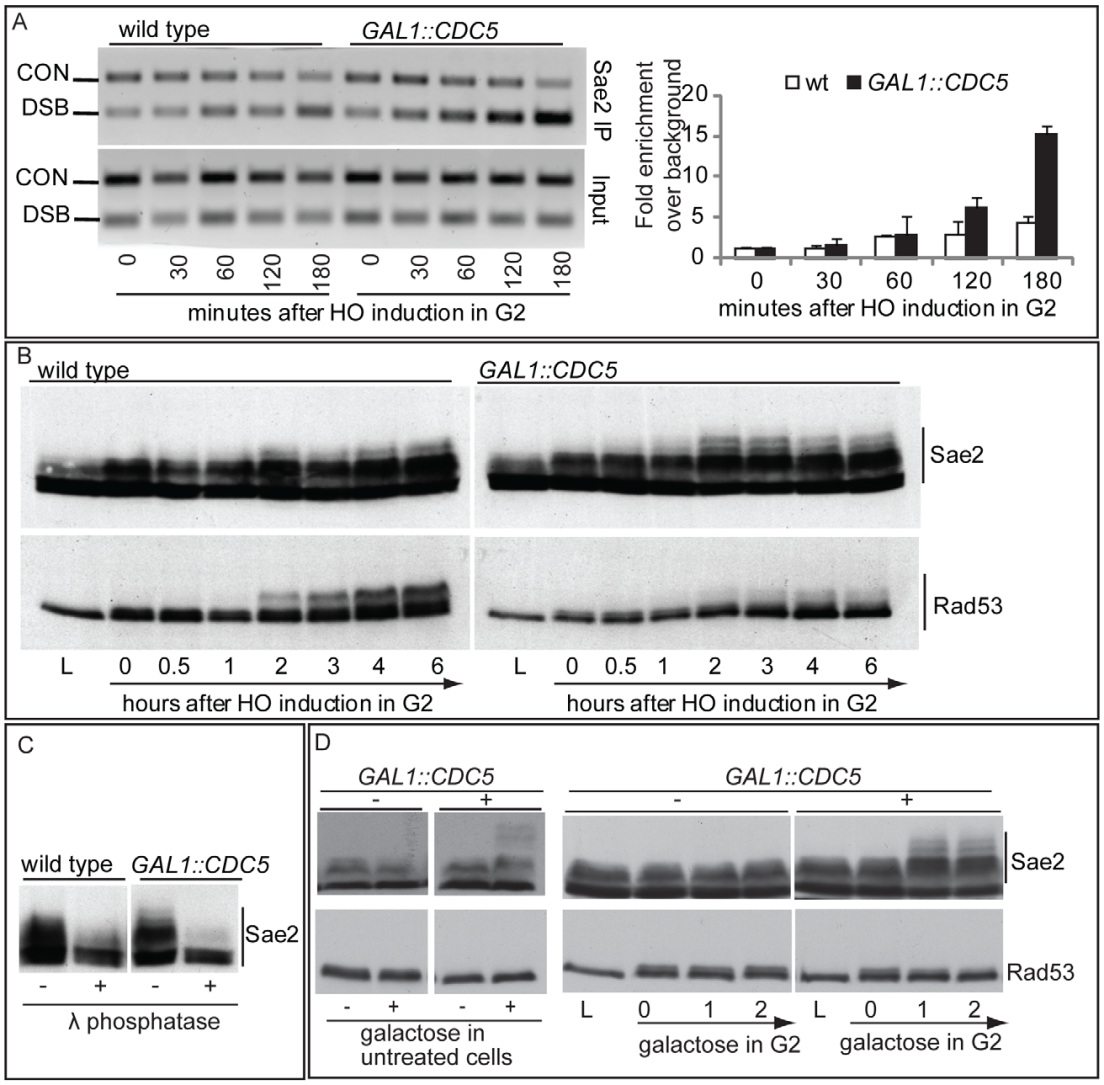
Analysis of Sae2 protein in *CDC5* overexpressing cells. (A,B) YEP+raffinose nocodazole-arrested cell cultures of wild type JKM and isogenic *GAL1::CDC5-MYC* strain, expressing *SAE2*-3HA allele, were transferred to nocodazole-containing YEP + raffinose + galactose (time zero). Cells were collected at the indicated times and then subjected to chromatin immunoprecipitation (ChIP) as described in Figure 6. Representative ChIP time-course analysis of protein-DSB association is shown before (Inputs) and after protein immunoprecipitation (IP). (B) Western blot analysis of protein extracts. (C) Western blot analysis of protein extracts prepared 3 hrs after HO induction and treated with or without λ phosphatase before gel electrophoresis. (D) YEP-raffinose growing cells of wild type and of wild-type JKM MATa-inc and isogenic *GAL1::CDC5-MYC* strains, expressing *SAE2*-3HA allele, were split in two. One half was treated with nocodazole to block cells in G2. Galactose was then added to the cultures to induce overproduction of Cdc5. Cells were collected at the indicated times after galactose addition. (B–D) Sae2-HA protein was analyzed by western blots using 12CA5 antibody; Rad53 protein was analysed by monoclonal antibody Mab.EL7.

**Figure 8:**
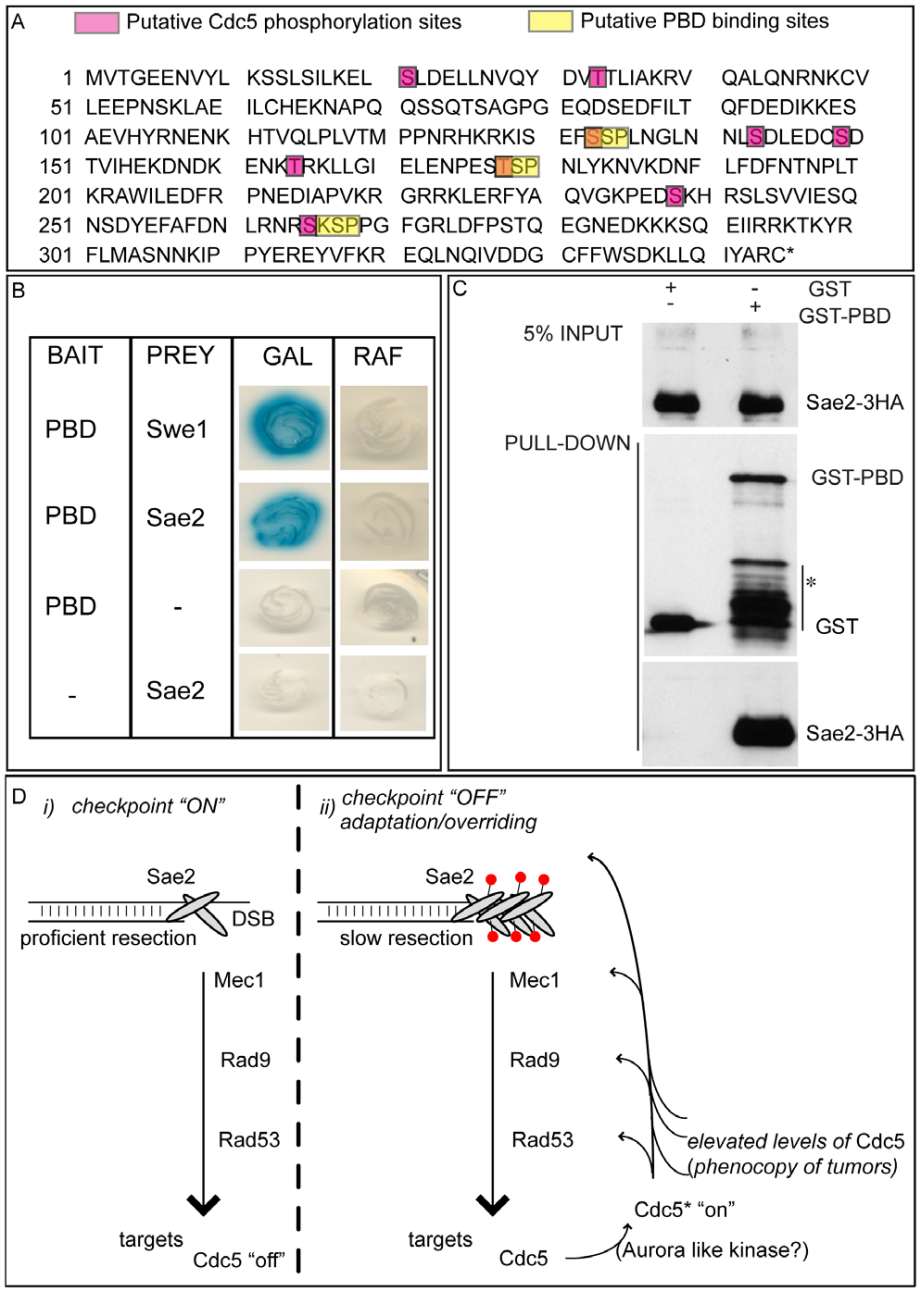
Sae2 protein interacts with PBD of Cdc5. (A) Sae2 protein sequence. The putative Cdc5 phosphorylation sites and PBD binding sites are indicated. (B) Plasmid pEG202-PBD_340–705_, carrying the polo box domain of Cdc5 (PBD, aa 340 to 705), and pJG4-5-SAE2, carrying the full length *SAE2* gene under the *GAL1* promoter, were co-transformed with pSH18-34, a β-galactosidase reporter plasmid in the wild type yeast strain EGY48. To assess two-hybrid interaction, these strains were patched on to 5-bromo-4-chloro-3-indolyl-β-D-galactopyranoside (X Gal) plates containing either raffinose (RAF, prey repressed) or galactose (GAL, prey expressed). Accordingly to [50], the strain Y692 (PBD versus Swe1_173–400_ protein fragment) was used as positive control. (C) Cells of the strain Y202, expressing *SAE2*-3HA gene, were blocked in G2/M by nocodazole treatment. Whole cell protein extract was prepared and incubated with glutathione-Sepharose beads carrying GST or GST-PBD_357–705_. Input and pull-down samples were analyzed by western blotting with monoclonal antibody 12CA5 (αHA) or polyclonal antisera raised against GST (αGST). Asterisk denotes bands of GST-PBD degradation or expression of truncated proteins. (D) Schematic model to summarize the results presented in this work. (i) Sae2 transiently binds DSB, regulating ends resection and influencing Mec1-signaling. The checkpoint signal is amplified downstream, regulating several targets, including Cdc5. (ii) After a prolonged checkpoint response, adaptation to damage takes over and Cdc5 is re-activated, likely by an activating kinase (in human cells, it is aurora A [35]); Cdc5 then inhibits checkpoint signaling in a feedback regulatory loop, by likely targeting several factors, including Sae2 whose loading on the irreparable DSB increases, slowing down resection and contributing to counteract the checkpoint signaling (red circles denote phosphorylation). Alternatively, or in addition, Cdc5 function on several targets, including Sae2, is enhanced in the presence of elevated levels of Cdc5, a situation frequently found for Plks in tumor cells.

We then tested the DSB binding of Sae2, which is a protein regulated by CDK1 [60] and PIKKs [65] after DNA damage and is involved in DSB processing [5] and checkpoint inactivation [66],[67]. Surprisingly, while in wild-type cells Sae2 loading was not significantly enriched at the HO-cut site (Figure 7A), likely because of its dynamic and transient binding to DSBs [67], Sae2 localization near the break greatly increased in *CDC5* overexpressing cells (Figure 7A). To test whether Cdc5 may specifically target Sae2 influencing its binding onto DSBs, we analysed the level and modification of Sae2 by western blotting following DSB formation. In nocodazole-blocked cells, induction of the HO cut caused PIKKs-dependent phosphorylation of Sae2 at the same time-points at which Rad53 phosphorylation was observed (Figure 7B). Interestingly, although high levels of Cdc5 impair Rad53 phosphorylation, they seem to cause hyperphosphorylation of Sae2. Infact, in *CDC5* overexpressing cells we observed the appearance of a ladder of slower migrating forms of Sae2 (Figure 7B), which are abolished by *in vitro* treatment with λ phosphatase (Figure 7C), indicating that they are due to phosphorylation events of Sae2. We then found that overproduction of Cdc5 induces Sae2 hyper-phosphorylation in untreated cells and in nocodazole-blocked cells without the HO-cut formation (Figures 7D), supporting the idea that Sae2 might be a direct target of Cdc5. Indeed, as mentioned above, Sae2 protein sequence reveals several sites that could be bound and/or phosphorylated by Cdc5 (Figure 8A). The C-terminus of Cdc5, like other Polo-like kinases, contains a phospho-serine/phospho-threonine binding domain called the Polo-box Domain (PBD) [19]. The PBD is known to bind Plk substrates after they have been “primed” by a preliminary phosphorylation by another protein kinase [19]. Interestingly, the putative PBD binding motif of Sae2 has been previously shown to be phosphorylated by CDK1 [60], making it a perfect candidate for mediating the interaction between Sae2 and Cdc5. Indeed, by a 2-hybrid assay we found that the PBD of Cdc5 interacts with Sae2 (Figure 8B), and a recombinant GST-PBD fusion protein, purified from *E. coli*, precipitated Sae2-3HA from yeast extracts (Figure 8C).

Taken together, the results shown in Figure 7 and Figure 8 indicate that Cdc5, through its PBD, interacts with Sae2, causing its hyper-phosphorylation and accumulation at the DSB (see also a model in Figure 8D). It is interesting to point out that CtIP, the functional ortholog of Sae2 in human cells, was found to be associated to chromatin following DNA damage and its chromatin binding is promoted by phosphorylation and ubiquitination [68]. Indeed, recent evidences indicate that CtIP and Ctp1 (the CtIP counterpart in *S. pombe* [69]), are recruited to DSB sites through their interaction with Nbs1 [70]–[72], a subunit of Mre11 complex, and BRCA1 [73],[74]. Moreover, CtIP is phosphorylated and regulated by CDK1 [74],[75]. In yeast, Sae2 is involved both in promoting an early step of DSB ends resection [5] and in inactivating checkpoint signaling during recovery and adaptation [66],[67], although the exact role of Sae2 in these processes is not yet fully understood. Interestingly, the overproduction of Sae2 also causes the overriding of the Mec1-signaling [66], while deletion of *SAE2* gene prevents switching off of the checkpoint [65],[66].

One possible working model (Figure 8D), which needs to be verified, predicts that the increased and persistent binding of Sae2 to a DSB, induced by overproduction of Cdc5, may affect both DSB resection and Mec1-signaling. It is tempting to speculate that even physiological levels of Cdc5 may regulate Sae2 during recovery and adaptation, contributing to switch off the checkpoint signal. It is also possible that Sae2 is regulated by Cdc5 only when this kinase is expressed at elevated levels, leading to the checkpoint overriding. Indeed, such situation is frequently observed in cancer cells, when Plks are overexpressed [36]–[39], suggesting that what we found in yeast may represent a model for a pathological condition in human cells. Future works, requiring the analysis of *sae2* mutations in the sites regulated by Cdc5, may help to discriminate between the two possibilities.

In conclusion, in the present study we further explored the role of the polo kinase Cdc5 in attenuating the DNA damage checkpoint in budding yeast. We found that overproduction of Cdc5 affects different parameters of the cellular response to an inducible DSB: i) it overrides Mec1 signaling and prevents the phosphorylation of various Mec1 targets (Rad53, Rad9, Ddc2); ii) it causes a slower resection of DSB ends in a *RAD9*-dependent manner; iii) it binds Sae2 protein, causing its hyper-phosphorylation and leading to its increased and persistent binding onto DSB.

The emerging scenario suggests that Cdc5 may target multiple factors involved in various aspects of the cellular response to DSB lesions and DNA damage checkpoint signaling. Indeed, Cdc5 is a fundamental regulator of cell cycle progression and targets many proteins throughout a normal cell cycle [20]. Most of the Cdc5 substrates are proteins previously phosphorylated by CDK1, which is the principal regulator of the DSB-induced response, regulating DSB processing, recombination and checkpoint signaling [57]. Here we found that high levels of Cdc5 separately affected Mec1 signaling and DSB processing, leading us to speculate that Cdc5 may regulate multiple targets in response to DNA damage, including factors phosphorylated by CDK1. In support of such hypothesis, Plks phosphorylate, in vertebrates, several proteins involved in various aspects of the DNA damage response, such as FANCM [25], Claspin [21]–[24], Chk2 [26]–[28], MCM5 [76], MCM7 [77] and others. Moreover, our findings on the functional role of Cdc5 in responding to a DSB in yeast rise the possibility that Plks may also regulate CtIP.

Recently, a proteome-wide screening led to the identification of novel Cdc5 targets in a normal cell cycle [20]; we believe that a similar approach is promising to identify Cdc5 targets regulated in response to DSBs. Good experimental evidence indicates that the functional role of Cdc5 in the DNA damage response is evolutionary conserved and the outputs of such a screening may provide important information for new cancer therapy strategies, targeting Plks and their substrates with specific tools.

## Materials and Methods

### Yeast strains and plasmids

Strains are listed in Table S1. All the strains were constructed during this study, and all were derivatives of JKM (*MATα*, *hmldelta::ADE1*, *hmrdelta::ADE1 ade1-100*, *trp1delta::hisG*, *leu2-3*, *leu2-112*, *lys5*, *ura3-52*, *ade3::GAL::HO*), with the exception of strain Y38, which was generated from strain Y5 (YMV80, *matΔ::hisG1*, *hmlΔ::ADE*, *hmrΔ::ADE1*, *lys5*, *ura3-52*, *leu2::HOcs*, *ade3::GAL::HO*, *his-URA3-5′Δleu2-is4*). To construct strains, standard genetic procedures for transformation and tetrad analysis were followed. Y38 and Y210 were obtained by integration of ApaI-digested plasmid pJC57 (p*GAL1*::*CDC5*-3HA) at the URA3 locus. Y215 was derived by integration of ApaI-digested pJC59 (p*GAL1*::*CDC5*-3myc) at *URA3* locus. Y220 was obtained by integration of ApaI-digested plasmid pJC62 (p*GAL1*::*cdc5*-K110A-3HA) at *URA3* locus. Y222 was obtained by integration of ApaI-digested plasmid pJC69 (p*GAL1*::cdc5-L251W-3HA) at *URA3* locus. Deletions and tag fusions were generated by the one-step PCR system [78]. The yeast two-hybrid assay was performed using the B42/lexA system with strain EGY48 (*Mata his3 ura3 trp1 6lexAOP*-*LEU2*; *lexAOP*-*lacZ* reporter on plasmid pSH18-34) as the host strain [79]. Bait plasmid pEG202-PBD_340–705_ for the two-hybrid assay, expressing lexA fusion with polo box domain of Cdc5, was obtained by amplifying the corresponding coding sequence of *CDC5* gene (aa 340 to 705) from genomic DNA and ligating the resulting fragment into pEG202 (kind gift from R. Brent). Prey plasmids pJG4-5-Swe1_173–400_ and pJG4-5-SAE2, expressing B42 activating domain fusions, were obtained by amplifying the corresponding coding sequence of *SWE1* (aa 173 to 400) and *SAE2* (full length) from genomic DNA and ligating the resulting fragments into pJG4-5.

### Western blot analysis

The TCA protein extraction and the western blot procedures have been previously described [29]. Rad53, Rad9, Sae2-HA, Ddc2-HA, Ddc1-myc, Dpb11-myc, Cdc5-HA, Cdc5-myc were analysed using specific monoclonal or polyclonal antibodies: anti-Rad53 Mab.EL7 and Mab.F9 monoclonal [54], anti-HA 12CA5 monoclonal, anti-myc 9E10 monoclonal, anti-Rad9 polyclonal (a kind gift from N Lowndes's lab).

### *In situ* auto-phosphorylation assay

It was performed as previously described [52].

### Immunoprecipitation analysis

Yeast whole cell extracts were prepared by FastPrep (MP Biomedicals) in NP-40 lysis buffer (1% NP-40, 150 mM NaCl, 50 mm Tris (pH 7.5), 1 mM dithiothreitol (DTT), 60 mM β-glycerophosphate, 1 mM NaVO_3_, cocktail proteases inhibitors (Roche)). HA-tagged proteins were immunoprecipitated using anti HA monoclonal antibody (12CA5) conjugated to protein G Agarose.

### GST pulldown assay

GST and GST-PBD were induced in BL21 *E. coli* cells as previously described [80] and conjugated to glutathione-Sepharose 4B beads (GSH beads, Amersham). Yeast whole cell extracts, prepared as indicated above, were incubated with GST or GST-PBD GSH beads and rotated for 1 hour at 4°C. Samples were washed three times with NP-40 buffer, boiled in SDS-based sample buffer, and analyzed by Western blotting analysis.

### In vitro dephosphorylation assay

Crude extracts were prepared as described [52], and resuspended in λ phosphatase buffer with or without 4000 U of λ phosphatase (Biolabs). Samples were incubated 30 min at 30°C and resuspended in Laemmli buffer.

### Measurements of DNA resection and SSA at DSBs

Cells grown in YEP-raffinose 3% medium at 28°C to a concentration of 5×10^6^ cells/ml were arrested with nocodazole (20µg/ml). A DSB was produced by adding 2% galactose and inducing the production of the HO endonuclease. The maintenance of the arrest was confirmed by FACS analysis and monitoring of nuclear division. Genomic DNA was isolated at intervals, and the loss of the 5′ ends of the HO-cleaved MAT locus was determined by Southern blotting [14],[81],[82]. To visualize the kinetics of resection, the graphs shown in Figure 4C and Figure 5B display, for each strain and for each ssDNA fragment (r1–r7), the time of the first appearance in the blot. In particular, since the appearance of a ssDNA fragment signal in the gel was due to the loss of the internal *Ssp*I sites, we represented the length of the minimal resection for each time point in the graph (see scheme in Figure 4A). All the experiments have repeated al least 3 times. In the corresponding figures, one representative example is shown with its graphic representation.

### Chromatin immunoprecipitation analysis (ChIP)

ChIP analysis was performed as described previously [83],[84]. Multiplex PCRs were carried out by using primer pairs complementary to DNA sequences located 1 kb from the HO-cut site at MAT (DSB) and to DNA sequences located 66 kb from MAT (CON). Gel quantitation was determined by using the NIH Image program. The relative fold enrichments of DSB-bound protein were calculated as follow: [DSB_IP/CON_IP]/[DSB_input/CON_input], where IP and Input represent the amount of PCR product in the immunoprecipitates and in input samples before immunoprecipitation, respectively.

## Supporting Information

Figure S1 Cellular levels of endogenous and overproduced Cdc5 protein. (A) Exponentially (L) growing culture of the strain Y79 (wild type) and Y114 (*GAL1::CDC5*) were grown in YEP+3%raffinose. The cell cultures were treated with nocodazole to block and maintained the cells in G2/M. Galactose was then added to induce the overproduction of Cdc5 and sample have been taken at the indicated times. (A) The cell cycle block in G2/M was analyzed by FACS. (B) Cdc5 protein was analysed by western blotting with polyclonal antibody, which recognized both the endogenous Cdc5 and the overproduced Cdc5-myc tagged protein.
(0.96 MB TIF)

Figure S2 Overproduced Cdc5 co-immunoprecipitates with Rad53. (A) Cultures of the strains Y79 (wild type), Y114 (*GAL1::CDC5*-MYC), exponentially (L) growing in YEP+3%raffinose were blocked in G2/M by nocodazole treatment (N) and zeocin (50 µg/ml) was then added to cause DSBs formation. After 30 min. of treatment with zeocin, 2% galactose was added and samples were taken after 1 hour. Overproduced Cdc5 protein has been immunoprecipitated with anti MYC antibody. Cdc5-MYC and Rad53 proteins were analysed by western blotting with monoclonal antibodies 9E10 (αMYC) and Ma.EL7 (αRad53).
(0.32 MB TIF)

Figure S3 Overproduction of Cdc5 does not prevent DSB repair by Single Strand Annealing (SSA). (A) Schematic representation of the YMV80 system used to detect DSB repair by SSA. Vertical bars show the relevant *KpnI* sites. After the HO cleavage, DNA is resected. When the left and right *leu2* sequences have been converted to ssDNA, repair by SSA can take place and can be monitored by the appearance of a SSA product in a Southern blot. (B,C) YEP+raffinose nocodazole-arrested cell cultures of wild type YMV80 and isogenic *GAL1::CDC5* strain were transferred to nocodazole-containing YEP+raffinose+galactose (time zero). (B) *KpnI*-digested genomic DNA, prepared from cells collected at the indicated times, was analysed by Southern blotting with a *LEU2* probe. Two fragments, 8 and 6 kb long (*his4::leu2*, *leu2::HOcs*) are evident in the absence of HO cut, whereas the HO-induced DSB causes the disappearance of the 6-kb species and the formation of a 2.5-kb fragment (HO-cut fragment). Repair by SSA converts such fragment to a repair product of 3.5-kb (SSA-product). (C) Western blot analysis of protein extracts with anti-Rad53 antibodies (Mab.EL7).
(0.81 MB TIF)

Table S1 Yeast strains used in this study.
(0.05 MB DOC)

## References

[1] Harrison JC, Haber JE (2006). Surviving the breakup: the DNA damage checkpoint.. Annu Rev Genet.

[2] Harper JW, Elledge SJ (2007). The DNA damage response: ten years after.. Mol Cell.

[3] Branzei D, Foiani M (2008). Regulation of DNA repair throughout the cell cycle.. Nat Rev Mol Cell Biol.

[4] Lazzaro F, Giannattasio M, Puddu F, Granata M, Pellicioli A (2009). Checkpoint mechanisms at the intersection between DNA damage and repair.. DNA Repair (Amst).

[5] Mimitou EP, Symington LS (2009). Nucleases and helicases take center stage in homologous recombination.. Trends Biochem Sci.

[6] Bartek J, Lukas J (2007). DNA damage checkpoints: from initiation to recovery or adaptation.. Curr Opin Cell Biol.

[7] Clemenson C, Marsolier-Kergoat MC (2009). DNA damage checkpoint inactivation: adaptation and recovery.. DNA Repair (Amst).

[8] Lee SE, Pellicioli A, Demeter J, Vaze MP, Gasch AP (2000). Arrest, adaptation, and recovery following a chromosome double-strand break in Saccharomyces cerevisiae.. Cold Spring Harb Symp Quant Biol.

[9] Galgoczy DJ, Toczyski DP (2001). Checkpoint adaptation precedes spontaneous and damage-induced genomic instability in yeast.. Mol Cell Biol.

[10] van Vugt MA, Medema RH (2004). Checkpoint adaptation and recovery: back with Polo after the break.. Cell Cycle.

[11] van de Weerdt BC, Medema RH (2006). Polo-like kinases: a team in control of the division.. Cell Cycle.

[12] Pellicioli A, Lee SE, Lucca C, Foiani M, Haber JE (2001). Regulation of Saccharomyces Rad53 checkpoint kinase during adaptation from DNA damage-induced G2/M arrest.. Mol Cell.

[13] Toczyski DP, Galgoczy DJ, Hartwell LH (1997). CDC5 and CKII control adaptation to the yeast DNA damage checkpoint.. Cell.

[14] Vaze MB, Pellicioli A, Lee SE, Ira G, Liberi G (2002). Recovery from checkpoint-mediated arrest after repair of a double-strand break requires Srs2 helicase.. Mol Cell.

[15] van Vugt MA, Bras A, Medema RH (2004). Polo-like kinase-1 controls recovery from a G2 DNA damage-induced arrest in mammalian cells.. Mol Cell.

[16] Syljuasen RG, Jensen S, Bartek J, Lukas J (2006). Adaptation to the ionizing radiation-induced G2 checkpoint occurs in human cells and depends on checkpoint kinase 1 and Polo-like kinase 1 kinases.. Cancer Res.

[17] Liu X, Lei M, Erikson RL (2006). Normal cells, but not cancer cells, survive severe Plk1 depletion.. Mol Cell Biol.

[18] Lane HA, Nigg EA (1996). Antibody microinjection reveals an essential role for human polo-like kinase 1 (Plk1) in the functional maturation of mitotic centrosomes.. J Cell Biol.

[19] Lowery DM, Mohammad DH, Elia AE, Yaffe MB (2004). The Polo-box domain: a molecular integrator of mitotic kinase cascades and Polo-like kinase function.. Cell Cycle.

[20] Snead JL, Sullivan M, Lowery DM, Cohen MS, Zhang C (2007). A coupled chemical-genetic and bioinformatic approach to Polo-like kinase pathway exploration.. Chem Biol.

[21] Peschiaroli A, Dorrello NV, Guardavaccaro D, Venere M, Halazonetis T (2006). SCFbetaTrCP-mediated degradation of Claspin regulates recovery from the DNA replication checkpoint response.. Mol Cell.

[22] Mamely I, van Vugt MA, Smits VA, Semple JI, Lemmens B (2006). Polo-like kinase-1 controls proteasome-dependent degradation of Claspin during checkpoint recovery.. Curr Biol.

[23] Mailand N, Bekker-Jensen S, Bartek J, Lukas J (2006). Destruction of Claspin by SCFbetaTrCP restrains Chk1 activation and facilitates recovery from genotoxic stress.. Mol Cell.

[24] Yoo HY, Kumagai A, Shevchenko A, Dunphy WG (2004). Adaptation of a DNA replication checkpoint response depends upon inactivation of Claspin by the Polo-like kinase.. Cell.

[25] Kee Y, Kim JM, D'Andrea AD (2009). Regulated degradation of FANCM in the Fanconi anemia pathway during mitosis.. Genes Dev.

[26] Bahassi el M, Conn CW, Myer DL, Hennigan RF, McGowan CH (2002). Mammalian Polo-like kinase 3 (Plk3) is a multifunctional protein involved in stress response pathways.. Oncogene.

[27] Tsvetkov LM, Tsekova RT, Xu X, Stern DF (2005). The Plk1 Polo box domain mediates a cell cycle and DNA damage regulated interaction with Chk2.. Cell Cycle.

[28] Petrinac S, Ganuelas ML, Bonni S, Nantais J, Hudson JW (2009). Polo-like kinase 4 phosphorylates Chk2.. Cell Cycle.

[29] Cheng L, Hunke L, Hardy CF (1998). Cell cycle regulation of the Saccharomyces cerevisiae polo-like kinase cdc5p.. Mol Cell Biol.

[30] Smits VA, Klompmaker R, Arnaud L, Rijksen G, Nigg EA (2000). Polo-like kinase-1 is a target of the DNA damage checkpoint.. Nat Cell Biol.

[31] van Vugt MA, Smits VA, Klompmaker R, Medema RH (2001). Inhibition of Polo-like kinase-1 by DNA damage occurs in an ATM- or ATR-dependent fashion.. J Biol Chem.

[32] Ando K, Ozaki T, Yamamoto H, Furuya K, Hosoda M (2004). Polo-like kinase 1 (Plk1) inhibits p53 function by physical interaction and phosphorylation.. J Biol Chem.

[33] Tsvetkov L, Stern DF (2005). Phosphorylation of Plk1 at S137 and T210 is inhibited in response to DNA damage.. Cell Cycle.

[34] Bassermann F, Frescas D, Guardavaccaro D, Busino L, Peschiaroli A (2008). The Cdc14B-Cdh1-Plk1 axis controls the G2 DNA-damage-response checkpoint.. Cell.

[35] Macurek L, Lindqvist A, Lim D, Lampson MA, Klompmaker R (2008). Polo-like kinase-1 is activated by aurora A to promote checkpoint recovery.. Nature.

[36] Lu LY, Yu X (2009). The balance of Polo-like kinase 1 in tumorigenesis.. Cell Div.

[37] Eckerdt F, Yuan J, Strebhardt K (2005). Polo-like kinases and oncogenesis.. Oncogene.

[38] Takai N, Hamanaka R, Yoshimatsu J, Miyakawa I (2005). Polo-like kinases (Plks) and cancer.. Oncogene.

[39] Smith MR, Wilson ML, Hamanaka R, Chase D, Kung H (1997). Malignant transformation of mammalian cells initiated by constitutive expression of the polo-like kinase.. Biochem Biophys Res Commun.

[40] Weichert W, Denkert C, Schmidt M, Gekeler V, Wolf G (2004). Polo-like kinase isoform expression is a prognostic factor in ovarian carcinoma.. Br J Cancer.

[41] Tokumitsu Y, Mori M, Tanaka S, Akazawa K, Nakano S (1999). Prognostic significance of polo-like kinase expression in esophageal carcinoma.. Int J Oncol.

[42] Knecht R, Oberhauser C, Strebhardt K (2000). PLK (polo-like kinase), a new prognostic marker for oropharyngeal carcinomas.. Int J Cancer.

[43] Kneisel L, Strebhardt K, Bernd A, Wolter M, Binder A (2002). Expression of polo-like kinase (PLK1) in thin melanomas: a novel marker of metastatic disease.. J Cutan Pathol.

[44] Yamada S, Ohira M, Horie H, Ando K, Takayasu H (2004). Expression profiling and differential screening between hepatoblastomas and the corresponding normal livers: identification of high expression of the PLK1 oncogene as a poor-prognostic indicator of hepatoblastomas.. Oncogene.

[45] Bartek J, Lukas J, Bartkova J (2007). DNA damage response as an anti-cancer barrier: damage threshold and the concept of ‘conditional haploinsufficiency’.. Cell Cycle.

[46] Sanchez Y, Bachant J, Wang H, Hu F, Liu D (1999). Control of the DNA damage checkpoint by chk1 and rad53 protein kinases through distinct mechanisms.. Science.

[47] Hu F, Wang Y, Liu D, Li Y, Qin J (2001). Regulation of the Bub2/Bfa1 GAP complex by Cdc5 and cell cycle checkpoints.. Cell.

[48] Song S, Grenfell TZ, Garfield S, Erikson RL, Lee KS (2000). Essential function of the polo box of Cdc5 in subcellular localization and induction of cytokinetic structures.. Mol Cell Biol.

[49] Song S, Lee KS (2001). A novel function of Saccharomyces cerevisiae CDC5 in cytokinesis.. J Cell Biol.

[50] Bartholomew CR, Woo SH, Chung YS, Jones C, Hardy CF (2001). Cdc5 interacts with the Wee1 kinase in budding yeast.. Mol Cell Biol.

[51] Charles JF, Jaspersen SL, Tinker-Kulberg RL, Hwang L, Szidon A (1998). The Polo-related kinase Cdc5 activates and is destroyed by the mitotic cyclin destruction machinery in S. cerevisiae.. Curr Biol.

[52] Pellicioli A, Lucca C, Liberi G, Marini F, Lopes M (1999). Activation of Rad53 kinase in response to DNA damage and its effect in modulating phosphorylation of the lagging strand DNA polymerase.. EMBO J.

[53] Pellicioli A, Foiani M (2005). Signal transduction: how rad53 kinase is activated.. Curr Biol.

[54] Fiorani S, Mimun G, Caleca L, Piccini D, Pellicioli A (2008). Characterization of the activation domain of the Rad53 checkpoint kinase.. Cell Cycle.

[55] Mantiero D, Clerici M, Lucchini G, Longhese MP (2007). Dual role for Saccharomyces cerevisiae Tel1 in the checkpoint response to double-strand breaks.. EMBO Rep.

[56] Toh GW, Lowndes NF (2003). Role of the Saccharomyces cerevisiae Rad9 protein in sensing and responding to DNA damage.. Biochem Soc Trans.

[57] Ira G, Pellicioli A, Balijja A, Wang X, Fiorani S (2004). DNA end resection, homologous recombination and DNA damage checkpoint activation require CDK1.. Nature.

[58] Lazzaro F, Sapountzi V, Granata M, Pellicioli A, Vaze M (2008). Histone methyltransferase Dot1 and Rad9 inhibit single-stranded DNA accumulation at DSBs and uncapped telomeres.. EMBO J.

[59] Ubersax JA, Woodbury EL, Quang PN, Paraz M, Blethrow JD (2003). Targets of the cyclin-dependent kinase Cdk1.. Nature.

[60] Huertas P, Cortes-Ledesma F, Sartori AA, Aguilera A, Jackson SP (2008). CDK targets Sae2 to control DNA-end resection and homologous recombination.. Nature.

[61] Kondo T, Wakayama T, Naiki T, Matsumoto K, Sugimoto K (2001). Recruitment of Mec1 and Ddc1 checkpoint proteins to double-strand breaks through distinct mechanisms.. Science.

[62] Zou L, Elledge SJ (2003). Sensing DNA damage through ATRIP recognition of RPA-ssDNA complexes.. Science.

[63] Lisby M, Barlow JH, Burgess RC, Rothstein R (2004). Choreography of the DNA damage response: spatiotemporal relationships among checkpoint and repair proteins.. Cell.

[64] Navadgi-Patil VM, Burgers PM (2009). A tale of two tails: Activation of DNA damage checkpoint kinase Mec1/ATR by the 9-1-1 clamp and by Dpb11/TopBP1.. DNA Repair (Amst).

[65] Baroni E, Viscardi V, Cartagena-Lirola H, Lucchini G, Longhese MP (2004). The functions of budding yeast Sae2 in the DNA damage response require Mec1- and Tel1-dependent phosphorylation.. Mol Cell Biol.

[66] Clerici M, Mantiero D, Lucchini G, Longhese MP (2006). The Saccharomyces cerevisiae Sae2 protein negatively regulates DNA damage checkpoint signalling.. EMBO Rep.

[67] Kim HS, Vijayakumar S, Reger M, Harrison JC, Haber JE (2008). Functional interactions between Sae2 and the Mre11 complex.. Genetics.

[68] Yu X, Fu S, Lai M, Baer R, Chen J (2006). BRCA1 ubiquitinates its phosphorylation-dependent binding partner CtIP.. Genes Dev.

[69] Limbo O, Chahwan C, Yamada Y, de Bruin RA, Wittenberg C (2007). Ctp1 is a cell-cycle-regulated protein that functions with Mre11 complex to control double-strand break repair by homologous recombination.. Mol Cell.

[70] Yuan J, Chen J (2009). N terminus of CtIP is critical for homologous recombination mediated double-strand break repair.. J Biol Chem.

[71] Lloyd J, Chapman JR, Clapperton JA, Haire LF, Hartsuiker E (2009). A supramodular FHA/BRCT-repeat architecture mediates Nbs1 adaptor function in response to DNA damage.. Cell.

[72] Williams RS, Dodson GE, Limbo O, Yamada Y, Williams JS (2009). Nbs1 flexibly tethers Ctp1 and Mre11-Rad50 to coordinate DNA double-strand break processing and repair.. Cell.

[73] Chen L, Nievera CJ, Lee AY, Wu X (2008). Cell cycle-dependent complex formation of BRCA1.CtIP.MRN is important for DNA double-strand break repair.. J Biol Chem.

[74] Yu X, Chen J (2004). DNA damage-induced cell cycle checkpoint control requires CtIP, a phosphorylation-dependent binding partner of BRCA1 C-terminal domains.. Mol Cell Biol.

[75] Huertas P, Jackson SP (2009). Human CtIP mediates cell cycle control of DNA end resection and double strand break repair.. J Biol Chem.

[76] Trenz K, Errico A, Costanzo V (2008). Plx1 is required for chromosomal DNA replication under stressful conditions.. EMBO J.

[77] Tsvetkov L, Stern DF (2005). Interaction of chromatin-associated Plk1 and Mcm7.. J Biol Chem.

[78] Longtine MS, McKenzie A, Demarini DJ, Shah NG, Wach A (1998). Additional modules for versatile and economical PCR-based gene deletion and modification in Saccharomyces cerevisiae.. Yeast.

[79] Gyuris J, Golemis E, Chertkov H, Brent R (1993). Cdi1, a human G1 and S phase protein phosphatase that associates with Cdk2.. Cell.

[80] Miller CT, Gabrielse C, Chen YC, Weinreich M (2009). Cdc7p-dbf4p regulates mitotic exit by inhibiting Polo kinase.. PLoS Genet.

[81] Lee SE, Moore JK, Holmes A, Umezu K, Kolodner RD (1998). Saccharomyces Ku70, mre11/rad50 and RPA proteins regulate adaptation to G2/M arrest after DNA damage.. Cell.

[82] Clerici M, Mantiero D, Lucchini G, Longhese MP (2005). The Saccharomyces cerevisiae Sae2 protein promotes resection and bridging of double strand break ends.. J Biol Chem.

[83] Viscardi V, Bonetti D, Cartagena-Lirola H, Lucchini G, Longhese MP (2007). MRX-dependent DNA damage response to short telomeres.. Mol Biol Cell.

[84] Clerici M, Mantiero D, Guerini I, Lucchini G, Longhese MP (2008). The Yku70-Yku80 complex contributes to regulate double-strand break processing and checkpoint activation during the cell cycle.. EMBO Rep.

