# Supplementary figures and images for: Elevated Levels of the Polo Kinase Cdc5 Override the Mec1/ATR Checkpoint in Budding Yeast by Acting at Different Steps of the Signaling Pathway
Source: PLoS Genet. 2010 Jan 22;6(1):e1000763. doi: 10.1371/journal.pgen.1000763 (PMC2797610; doi:10.1371/journal.pgen.1000763)

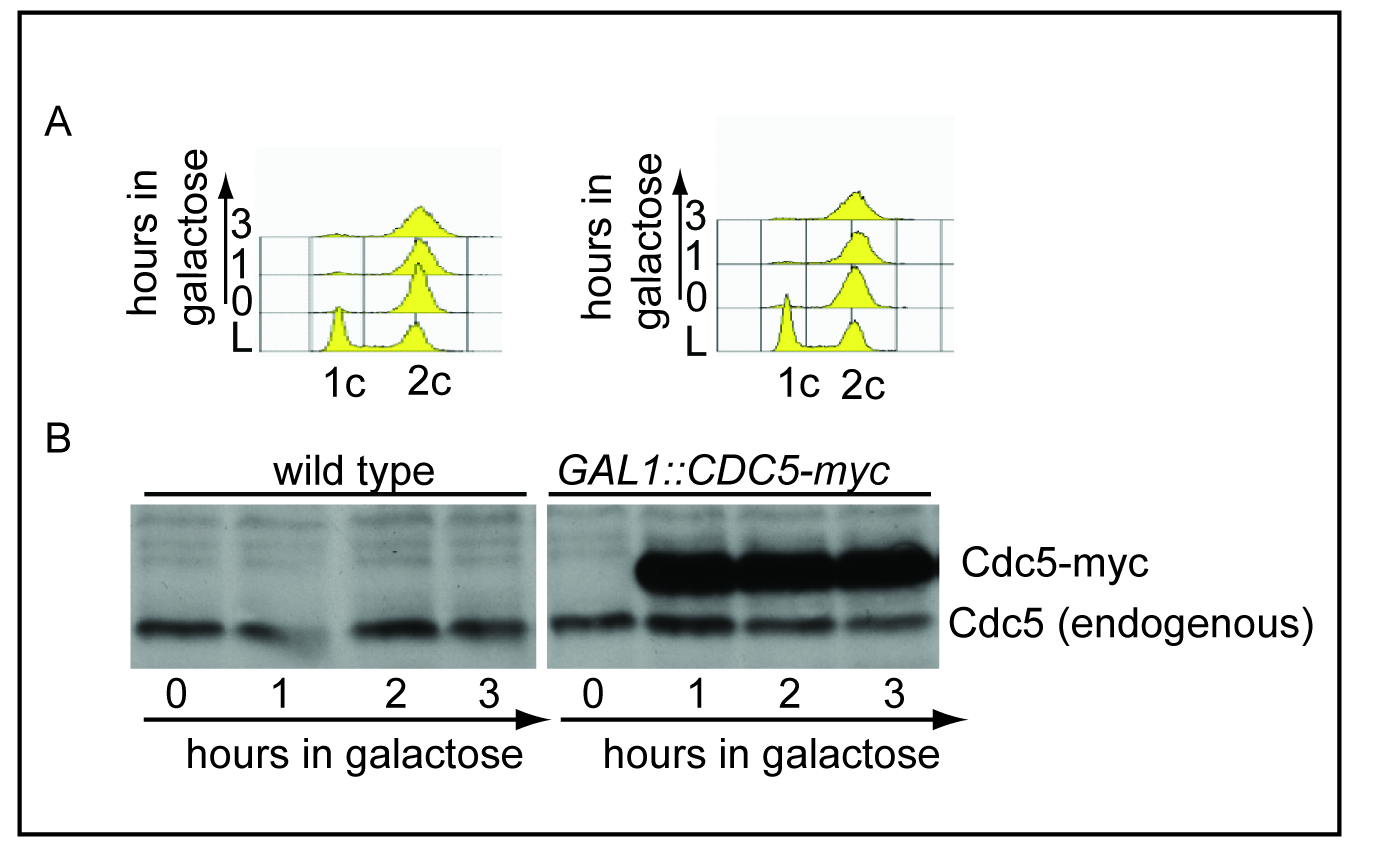

Supplement: Figure S1 — Cellular levels of endogenous and overproduced Cdc5 protein. (A) Exponentially (L) growing culture of the strain Y79 (wild type) and Y114 (GAL1::CDC5) were grown in YEP+3%raffinose. The cell cultures were treated with nocodazole to block and maintained the cells in G2/M. Galactose was then added to induce the overproduction of Cdc5 and sample have been taken at the indicated times. (A) The cell cycle block in G2/M was analyzed by FACS. (B) Cdc5 protein was analysed by western blotting with polyclonal antibody, which recognized both the endogenous Cdc5 and the overproduced Cdc5-myc tagged protein. (0.96 MB TIF) [file pgen.1000763.s001.tif]

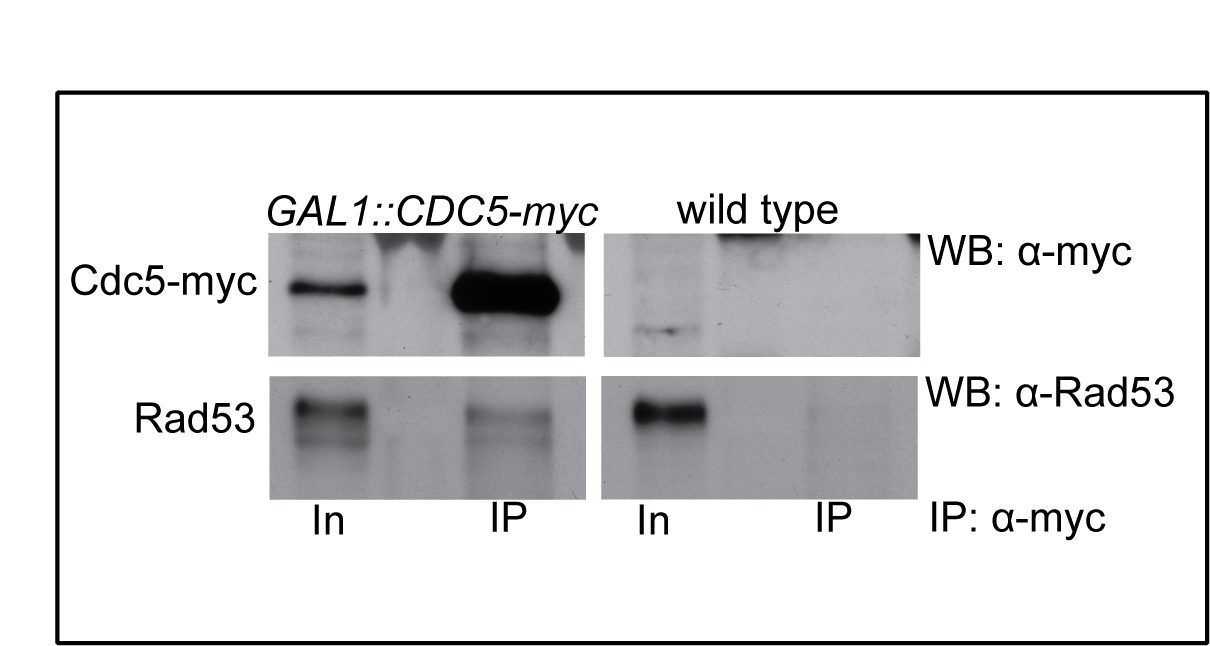

Supplement: Figure S2 — Overproduced Cdc5 co-immunoprecipitates with Rad53. (A) Cultures of the strains Y79 (wild type), Y114 (GAL1::CDC5-MYC), exponentially (L) growing in YEP+3%raffinose were blocked in G2/M by nocodazole treatment (N) and zeocin (50 µg/ml) was then added to cause DSBs formation. After 30 min. of treatment with zeocin, 2% galactose was added and samples were taken after 1 hour. Overproduced Cdc5 protein has been immunoprecipitated with anti MYC antibody. Cdc5-MYC and Rad53 proteins were analysed by western blotting with monoclonal antibodies 9E10 (αMYC) and Ma.EL7 (αRad53). (0.32 MB TIF) [file pgen.1000763.s002.tif]

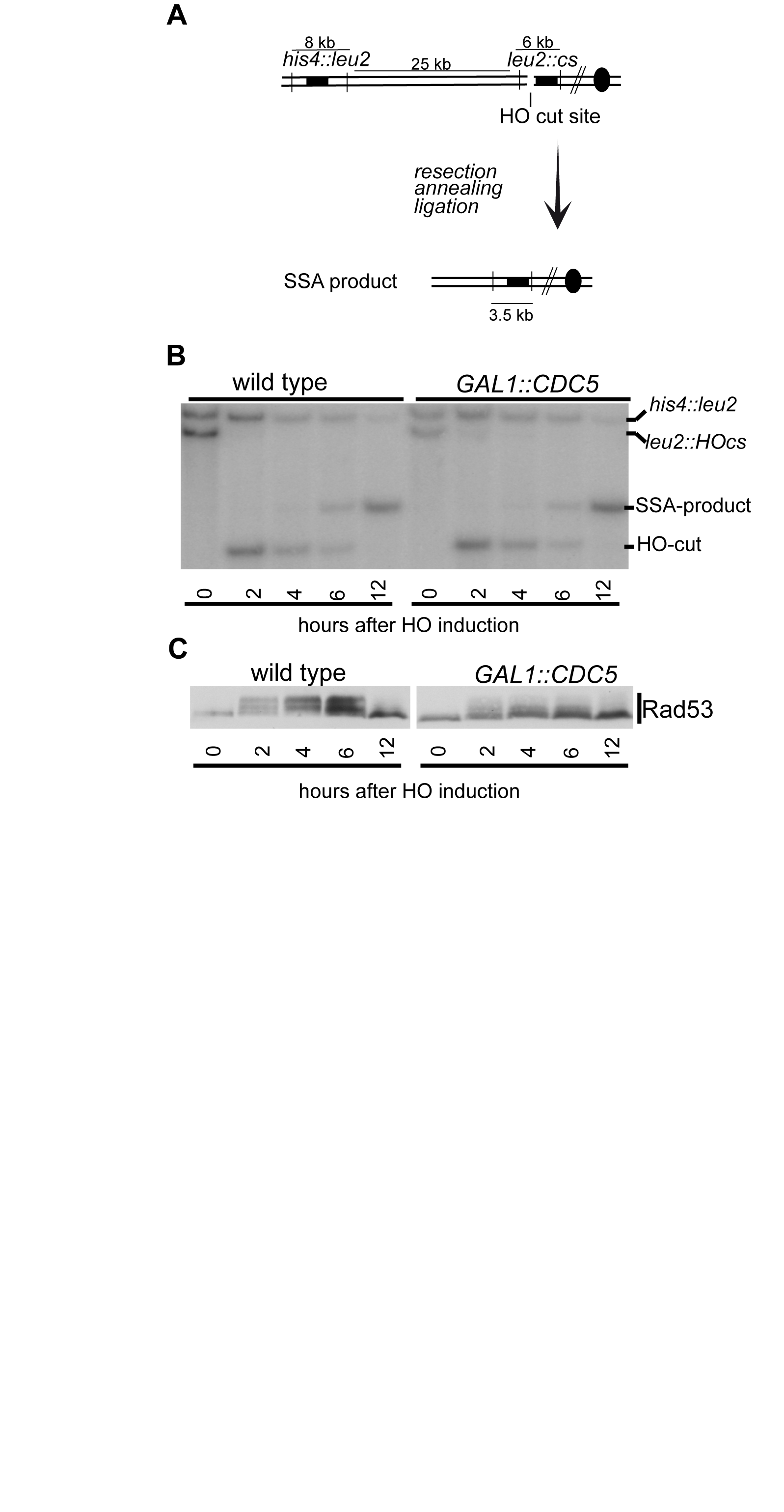

Supplement: Figure S3 — Overproduction of Cdc5 does not prevent DSB repair by Single Strand Annealing (SSA). (A) Schematic representation of the YMV80 system used to detect DSB repair by SSA. Vertical bars show the relevant KpnI sites. After the HO cleavage, DNA is resected. When the left and right leu2 sequences have been converted to ssDNA, repair by SSA can take place and can be monitored by the appearance of a SSA product in a Southern blot. (B,C) YEP+raffinose nocodazole-arrested cell cultures of wild type YMV80 and isogenic GAL1::CDC5 strain were transferred to nocodazole-containing YEP+raffinose+galactose (time zero). (B) KpnI-digested genomic DNA, prepared from cells collected at the indicated times, was analysed by Southern blotting with a LEU2 probe. Two fragments, 8 and 6 kb long (his4::leu2, leu2::HOcs) are evident in the absence of HO cut, whereas the HO-induced DSB causes the disappearance of the 6-kb species and the formation of a 2.5-kb fragment (HO-cut fragment). Repair by SSA converts such fragment to a repair product of 3.5-kb (SSA-product). (C) Western blot analysis of protein extracts with anti-Rad53 antibodies (Mab.EL7). (0.81 MB TIF) [file pgen.1000763.s003.tif]
