# Supplementary material for: Elevated Levels of the Polo Kinase Cdc5 Override the Mec1/ATR Checkpoint in Budding Yeast by Acting at Different Steps of the Signaling Pathway
Source: PLoS Genet. 2010 Jan 22;6(1):e1000763. doi: 10.1371/journal.pgen.1000763 (PMC2797610; doi:10.1371/journal.pgen.1000763)
Supplement: Table S1 — Yeast strains used in this study. (0.05 MB DOC) [file pgen.1000763.s004.doc]

TABLE 1 *S. cerevisiae* strains used in this study

| Strain | Relevant genotype | Reference/source |
| --- | --- | --- |
| Y1 | *MATα, hmldelta::ADE1, hmrdelta::ADE1 ade1-100, trp1delta::hisG, leu2-3, leu2-112, lys5, ura3-52, ade3::GAL::HO* | J Haber’s laboratory |
| Y5 | *matΔ::hisG1, hmlΔ::ADE, hmrΔ::ADE1, lys5, ura3-52, leu2::HOcs , ade3::GAL::HO, his-URA3-5'Δleu2-is4* | J Haber’s laboratory |
| Y34 | *Y1 rad53-K227A-KanMX6* | This study |
| Y38 | *Y5 <GAL1::CDC5-3HA@URA3>* | This study |
| Y79 | *MATa-inc, hmldelta::ADE1, hmrdelta::ADE1 ade1-100, trp1delta::hisG, leu2-3, leu2-112, lys5, ura3-52, ade3::GAL::HO* | J Haber’s laboratory |
| Y114 | *Y79<GAL1::CDC5-3myc@URA3>* | This study |
| Y117 | *MATa, hmldelta::ADE1, hmrdelta::ADE1 ade1-100, trp1delta::hisG, leu2-3, leu2-112, lys5, ura3-52, ade3::GAL::HO* | This study |
| Y184 | *Y79<GAL1::cdc5-K110A-3HA-3HA@URA3>* | This study |
| Y185 | *Y79<GAL1::cdc5-L251W-3HA@URA3>* | This study |
| Y202 | *Y117 SAE2-3HA-URA3* | This study |
| Y210 | *Y117 DDC2-3HA-URA3* | This study |
| Y215 | *Y210 <GAL1::CDC5-3myc@URA3>* | This study |
| Y220 | *Y117 <GAL1::cdc5-K110A-3HA-3HA@URA3>* | This study |
| Y222 | *Y117 <GAL1::cdc5-L251W-3HA@URA3>* | This study |
| Y567 | *EGY48<pSH18-34><pEG202><pJG4-5-SAE2>* | This study |
| Y670 | *Y1 RAD9-13myc-TRP1* | This study |
| Y674 | *Y670 <GAL1::CDC5-3HA@URA3>* | This study |
| Y677 | *Y34 <GAL1::CDC5-3HA@URA3>* | This study |
| Y684 | *EGY48<pSH18-34><pEG202-PBD><pJG4-5>* | This study |
| Y690 | *EGY48<pSH18-34><pEG202-PBD><pJG4-5-SAE2>* | This study |
| Y692 | *EGY48<pSH18-34><pEG202-PBD><pJG4-5-SWE1_173-400_>* | This study |
| Y754 | *Y117 DDC1-13myc-KanMX6* | This study |
| Y757 | *Y754 <GAL1::CDC5-3HA@URA3>* | This study |
| Y790 | *Y117 DPB11-13myc-KanMX6* | This study |
| Y820 | *Y790 <GAL1::CDC5-3HA@URA3>* | This study |
| Y830 | *Y1 rad9::KanMX6 DDC2-HA-URA3 <GAL1::CDC5-3myc@URA3>* | This study |
| Y839 | *Y202 <GAL1::CDC5-3myc@URA3>* | This study |
| Y834 | *Y1 rad9::KanMex6 DDC2-3HA-URA3* | This study |
